# Physicochemical Properties of Biobutanol as an Advanced Biofuel

**DOI:** 10.3390/ma14040914

**Published:** 2021-02-15

**Authors:** Michal Obergruber, Vladimír Hönig, Petr Procházka, Viera Kučerová, Martin Kotek, Jiří Bouček, Jakub Mařík

**Affiliations:** 1 Department of Chemistry, Faculty of Agrobiology, Food and Natural Resources, Czech University of Life Sciences Prague, Kamýcká 129, 169 21 Prague 6, Czech Republic; obergruber@af.czu.cz; 2 Department of Economics, Faculty of Economics and Management, Czech University of Life Sciences Prague, Kamýcká 129, 169 21 Prague 6, Czech Republic; pprochazka@pef.czu.cz; 3 Department of Chemistry and Chemical Technology, Faculty of Wood Sciences and Technology, Technical University of Zvolen, 960 53 Zvolen, Slovakia; viera.kucerova@tuzvo.sk; 4 Department of Vehicles and Ground Transport, Faculty of Engineering, Czech University of Life Sciences Prague, Kamýcká 129, 169 21 Prague 6, Czech Republic; kotekm@oikt.czu.cz (M.K.); marikj@tf.czu.cz (J.M.); 5 Department of Applied Ecology, Faculty of Environmental Sciences, Czech University of Life Sciences Prague, Kamýcká 129, 165 21 Praha 6, Czech Republic; jboucek@fld.czu.cz

**Keywords:** butanol, ethanol, biomaterials, alternative fuel, second generation, distillation, octane number, vapor pressure

## Abstract

Biobutanol is a renewable, less polluting, and potentially viable alternative fuel to conventional gasoline. Biobutanol can be produced from same sources as bioethanol, and it has many advantages over the widespread bioethanol. This paper systematically analyzes biobutanol fuel as an alternative to bioethanol in alcohol–gasoline mixtures and the physicochemical properties. Based on the conducted analyses, it was found that biobutanol mixtures have a more suitable behavior of vapor pressure without the occurrence of azeotrope, do not form a separate phase in lower temperature, it has higher energy density, but slightly reduce the octane number and a have higher viscosity. However, in general, biobutanol has many advantageous properties that could allow its use in gasoline engines instead of the commonly used bioethanol.

## 1. Introduction

The current global situation of depleting oil reserves leads to a society-wide acceptance of the fact that it is necessary to modernize the way of obtaining fuels and energy. Climate change and energy security are at the forefront of nations’ interests, influencing social, economic, and political decisions. Therefore, there is a need to focus more on the research and development of innovative energy alternatives that have the potential to prevent problems with global change and energy security [1,2].

Hence, researchers have turned their attention to biofuels, viable alternatives to oil, and other fuel products. Biofuels have a number of benefits that can accelerate the adaptation of biofuels worldwide. This opens up the possibility of sustainable and renewable fuels, lowers the dependence on imported fossil fuels, exhaust less pollutants, like NO_x_, CO, CO_2_, HC, or PM [3,4,5,6,7], and expands the global market for agricultural products [8,9]. The production of biofuels will also improve the economic position of local workers by creating jobs [10]. Biofuels of the first generation, i.e., bioethanol and biodiesel that are produced from food sources, are currently the most common alternative fuel. Because the source material for these fuels can also be used in the production of food and feed, there is a competitive use for them, which cannot be sustainable in the long run. Therefore, it can affect the reduction of food supply and increase food prices [11,12].

Because to this conflict between food and fuels, attention is beginning to turn to waste biomass, non-food biomass, and waste available as a by-product of other processes that can also be used for energy purposes and do not compete with food sources. By processing them, it is possible to obtain biogas, biodiesel, and bioalcohols of the same quality. This is also the general difference between bioethanol and ethanol, or generally bioalcohol and alcohol. When the compound is derived from, e.g., oil, it is usually referred to simply as “ethanol” or generally “alcohol” and, when it is obtained from biological matter or plant sources, it can be referred as “bioethanol” or generally “bioalcohol”. Otherwise, they both have the same chemical composition and have same physicochemical properties. Fuels that are produced from waste or by-products are not affecting the food supply, together with other positive effects, such as reducing emissions of pollutants, supporting the circular economy, reducing dependence on external sources, and reducing landfilling [13]. These fuels are called biofuels of the second generation.

The biofuels of the second generation that are produced from plant biomass mainly concern lignocellulosic materials, as most are cheap and commonly available nonfood plant materials. Composition (cellulose, hemicellulose, and lignin) of different lignocellulosic materials can be found, for example, in [14]. Cellulose is the main component of this biomass (making up about 45% of the dry weight of wood) and it consists of a polymer of D-glucose linked by a β‑1,4‑glycosidic bond to form cellobiose molecules [15]. Hemicellulose is a copolymer that is composed of various pentoses and hexoses and uronic acids. Common sugars that are found in hemicellulose are xylose, arabinose, mannose, or galactose, with 50–200 units. Xylan is the main component of agricultural biomass and hardwood hemicellulose, while glucomannan is the main component of softwood [16]. Lignin is a copolymer of phenyl propionic alcohol units, i.e., paracoumaryl alcohol, sinapyl alcohol, and coniferyl alcohol, which are linked to each other by carbon–carbon (C–C) and ether–carbon (C–O) bonds. The location of lignin is between the cellulose and hemicellulose that bind together [16,17]. Other source of organic material can be synthesis gas or syngas. Syngas is produced by the gasification of organic compounds by partial oxidation. Syngas contains ~30% (V/V) of carbon monoxide, 25–30% (V/V) of hydrogen, 20–30% (V/V) of carbon dioxide, ~10% (V/V) of methane, and 3% (V/V) of ethane [18]. Syngas quality largely depends on the composition of feedstock, gasifier type, and the gasifying agents.

Syngas can be transformed to alcohols by several ways. The use of catalysts is one of the categories. These can be generally classified into four categories: Noble metal (rhodium) based catalyst [19], modified methanol synthesis catalysts [20], modified Fischer-Tropsch synthesis catalysts [21], and molybdenum-based catalyst [22]. Luk et al. compiled an overview of the state-of-the-art of this catalyst family [23]. The significant downsides of catalyst synthesis are the cost of metal catalysts, the potential of catalyst poisoning due to inert gases and contaminants (e.g., sulfur), and high operating temperature and pressure [24,25,26]. Moreover, the synthesis requires consistent gas quality and a H_2_/CO ratio to produce the desired products.

Instead of synthesis gas catalysis, another approach can be used. Syngas fermentation is carried out by microbial catalysts that are known as homo-acetogenic, such as *Clostridium ljungdahlii*, *Clostridium carboxidivorans, Clostridium autoethanogenum, Eubacterium limosum, Clostridium ragsdalei*, and *Alkalibaculum bacchi* [27,28,29]. When compared to chemical catalysts, syngas fermentation has the advantage of high specificity to the substrate, operation at low temperature and pressure, and high tolerance to toxic gases [30,31]. Syngas fermentation is normally operated at ambient pressure, 101 kPa, and 37 °C to favor the microorganism growth condition [31,32,33]. Syngas fermentation utilizes different H_2_/CO ratios that are generated from the gasifier [27,34,35]. The impurities in syngas (NH_3_, H_2_S, SO_x_, COS, HCN, and HCl) that are generated during gasification are better tolerated by microorganisms than catalysts [34,36,37].

Although the most common biofuel added to gasoline is bioethanol, researchers are increasingly focusing on advanced biofuels and more complex alcohols. One of the promising advanced biofuels with better fuel properties than ethanol is proved to be butanol [38,39,40,41,42,43,44,45,46,47].

Butanol has four isomeric structures depending on the position of the hydroxyl (–OH) group on the carbon chain: n–butanol (1–butanol), s–butanol (2–butanol), i–butanol (2–methyl–1–propanol), and t–butanol (2–methyl–2–propanol). 

In the production of butanol from agricultural raw materials, n-butanol and i–butanol are preferred. Out of all the isomers, n–butanol was most often studied as a motor fuel [48]; however, the development of processes for the production of i–butanol from biomass sources is an active area of research and its potential as a fuel is gaining interest [49,50,51].

The most common n–butanol-producing organisms are members of the genus *Clostridium* by Acetone-Butanol-Ethanol (ABE) fermentation. There are several species of *Clostridium* that can produce butanol, including *C. acetobutylicum, C. saccharoperbutylacetonicum, C. beijerinckii, C. saccharoacetobutylicum, C. cadaversis, C. sporogenes, C. pasteurianum,* and *C. tetanomorphum* [12]. The first four above-mentioned species have been shown to produce the highest volumes of butanol [52]. ABE fermentation converts sugar into butanol, acetone, and ethanol in a ratio of 6:3:1, respectively [53].

The methods of producing any bioalcohol is an important topic in these days. The European Union committed that all engine fuels that are distributed in the EU market will contain a certain share of biocomponent. Gasoline is the second most used motor fuel in the European Union after diesel [54,55], and this ratio is also reflected in the consumption of these biocomponents. The most used biofuel is the biodiesel with 71.98% of total consumption, followed by bioethanol with 21.90%. The quality requirements and parameters of motor gasolines are described in the European standard EN 228 [56]. According to Directive 2009/30/EC [57], Member States shall require suppliers to ensure the placing on the market of gasoline with a maximum oxygen content of 2.7%. Which effectively means 7.3% (V/V) of ethanol but up to 11.7% (V/V) of butanol. At present, ethanol is mixed only at a maximum of 5% (V/V). In 2015, the EU founded a project Horizon 2020 ButaNexT in order to examine the potential of biobutanol, which investigated the possibility of the production and use of the biobutanol in the EU [58].

As already mentioned, butanol has more favorable properties when compared to ethanol. Butanol contains 25% more energy than ethanol, which, in unmodified powertrains, means a reduction in fuel consumption, which is higher in the case of ethanol. Gasoline engines can burn mixtures of gasoline with butanol in any ratio of these two components, but butanol can also be separately used in pure form. It is not as hygroscopic as ethanol, so it is less corrosive to metal tanks and pipes, nor does a separate phase of water with butanol (it behaves similarly to MTBE or ETBE). This property further extends its versatility, because it can be supplied with the currently used fuel pipes while ethanol needs railway transport. In terms of usage, it is safer than ethanol due to lower vapor pressure. The volatility (=Reid vapour pressure) of n-butanol is approx. 7.5 times lesser than ethanol and approx. 32 times lesser than gasoline. During combustion, butanol can emit lower concentrations of SO_x_ and NO_x_. The properties of butanol are closer to gasoline than ethanol and methanol. For high butanol and low gasoline fuel blends, the technical changes that are required to the vehicle’s original gasoline fuel system are less extensive than for high ethanol blends. Table 1 provides the different properties of butanol and some conventional fuels [59].

Based on the discussions above, as a renewable fuel, butanol has great potential for application in compression ignition engines. The aim of the article is to design and verify the possibility of using butanol as a fuel in gasoline engines and, thus, show a more suitable use of biomaterials that are used for fuel production. The comprehensive analysis contains the measurement of the physicochemical properties of fuel mixtures, fuel characteristics, and evaluation of applicability.

## 2. Materials and Methods

Mixtures with working names were selected in order to determine the effect of n-butanol, i–butanol, and ethanol on gasoline:

- Ex: x% (V/V) ethanol and (100–x)% (V/V) gasoline (e.g., E5).
- BUT x:x% (V/V) n–butanol and (100–x)% (V/V) gasoline (e.g., BUT 5).
- iBUT x:x% (V/V) i–butanol and (100–x)% (V/V) gasoline (e.g., iBUT 5).
- BUT x + MTBE y: x% (V/V) n–butanol, y% (V/V) methyl tert–butyl ether, and (100–x–y)% (V/V) gasoline (e.g., BUT 5 + MTBE 10).
- BUT x + ETBE y: x% (V/V) n–butanol, y% (V/V) ethyl tert-butyl ether, and (100–x–y)% (V/V) gasoline (e.g., BUT 5 + ETBE 10).

Pure gasoline was produced by Unipetrol RPA and it is fully compliant with standard EN 228 for winter class (F1). It contained 32.21% (V/V) of aromatic hydrocarbons, 10.29% (V/V) of olefins, and 0.52% (V/V) of benzene. The water content was 48.00 mg/kg and the oxidative stability exceeded 360 min. Methyl tert-butyl ether (MTBE) AR (Analytical Reagent. High grade of purity) and ethyl tert-butyl ether AR (ETBE) were also produced from Unipetrol RPA. n-Butanol AR, and i-butanol AR were produced by LachNer, s.r.o. The tested ethanol for comparison fully complied with the requirements of EN 15376:2014 standard [64].

The physiochemical properties of alcohol were determined in order to identify the fuel properties of the mixtures. In the evaluation of fuel density, kinematic viscosity, octane number, Reid vapor pressure, water solubility, and distillation curves were measured. These fuel properties were compared for fuels containing volumetric amounts of different alcohols.

An analytical method for the determination of ethanol, i–butanol, and n–butanol in gasoline using Gas Chromatography with Flame-Ionization Detection (GC-FID) was also validated and conducted. Chromatography is a physical method of separation, in which the components that are to be separated are distributed between two phases, one of which is fixed (stationary phase), while the other (the mobile phase) moves in a definite direction. A mobile phase is described as “a fluid that percolates through or along the stationary bed in a definite direction”. It may be a liquid, gas, or supercritical fluid, while the stationary phase may be a solid, a gel, or a liquid. If a liquid, it may be distributed on a solid, which may or may not contribute to the separation process [65].

GC analysis were carried out while using the gas chromatograph Varian 3300 (Varian, Walnut Creek, CA, USA) that was equipped with a fused silica capillary column DB-5 (30 m × 0.25 mm I. D., film thickness 0.25 μm) and a flame ionization detector (FID) where hydrogen (30 mL/min.) in air (300 mL/min.) was used. The column temperature program was 50 °C for 3 min., gradient 8 °C/min., upper isotherm 260 °C for 5 min.; injection port and detector temperature 260 °C, split ratio 1:20, carrier gas nitrogen (flow 1 mL/min.).

The volume percentage was calculated from the measured quantities of the individual substances before mixing. The samples were stored in dark brown glass bottles with a volume of 20 mL. For GC measurement, the test samples were dissolved in isooctane and nonane was added to all samples as an internal standard. The samples were mixed according to the following scheme: 1000 µL of isooctane + 10 µL of gasoline + 10 µL of nonane. The diluted samples with a suitable solvent, in this case isooctane, have improved the separation efficiency of the chromatographic column.

An evaluation was carried out by an internal standard. Hydrocarbon nonane was used as the internal standard. Calibration curves for n-butanol and i–butanol were measured using standard solutions of these substances in isooctane at three concentration levels (in each of the three vials were 1000 µL of isooctane + 2 µL or 5 µL or 10 µL of alcohols + 10 µL of nonane).

All of the parameters were always measured three times and the results represent the average value from three measurements with the expanded uncertainty with 95% confidence interval. The expanded uncertainty *U* of the measurand was obtained by multiplying the combined standard uncertainty *u*(*y*) by a coverage factor *k*, which gives the best estimate of the value attributable to the measurand. The value of the coverage factor k was chosen to meet the probability of coverage of about 95%, which, for a normal distribution, corresponds to the factor *k* = 2 [66].

The density was determined according to the standard ISO 3675:1998–Crude petroleum and liquid petroleum products–Laboratory determination of density–Hydrometer method [67]. The kinematic viscosity was determined according to the standard ISO 3104:1994 Petroleum products–Transparent and opaque liquids–Determination of kinematic viscosity and calculation of dynamic viscosity [68]. The research octane number was determined according to the standard ISO 5164:2014–Petroleum products–Determination of knock characteristics of motor fuels–Research method [69]. The Reid vapor pressure was determined according to the standard ISO 3007:1999–Petroleum products and crude petroleum–Determination of vapor pressure–Reid method [70]. The water solubility was determined according to the standard ASTM D6422-99-Test Method for Water Tolerance (Phase Separation) of Gasoline-Alcohol Blends [71]. The distillation curve of the mixture was determined according to the standard ISO 3405:2011–Petroleum products–Determination of distillation characteristics at atmospheric pressure [72]. The gas chromatography was performed according to the standard ISO 22854:2016–Liquid petroleum products–Determination of hydrocarbon types and oxygenates in automotive-motor gasoline and in ethanol (E85) automotive fuel–Multidimensional gas chromatography method [73].

Matlab R2015a (MathWorks, Natick, MA, USA) and R 4.0.2 (R Core Team) were used for the statistical evaluation and graphical representation of the results. The data collection software used in GC was Star Chromatography Workstation vs. 4.51 (Varian, Walnut Creek, CA, USA).

## 3. Results

### 3.1. Fuel Parameters

Table 2 gives the measured values of the fuel parameters of the mixtures and they represent the average value from three measurements. The density values did not differ in three measurements; the expanded uncertainty of the result determination is ±0.5 kg/m^3^ of the result value. The distillation temperatures of the given sample from three measurements differed by less than 2 °C. The extended uncertainty of measurement is ±4 °C and ±2% (V/V). The Reid vapor pressure values from three measurements did not differ. The extended uncertainty of the result determination is ±1%. The kinematic viscosity values from three measurements differed by less than 1 s with the flow through the viscometer, the expanded uncertainty of the result determination is ±1% of the result value. The flash point temperatures from three measurements differed by less than 1 °C. The expanded uncertainty of the result determination is ±1 °C.

Table 2 shows the influence of butanol on the distillation process and vapor pressure of gasoline. These parameters are reflected in the reduction of the volatility index.

The research octane number of ethanol and butanol from three measurements did not differ. The expanded uncertainty of the result determination is ±0.4 units of research octane number. The correction factor of 0.2 was subtracted from the results.

**Figure 1:**
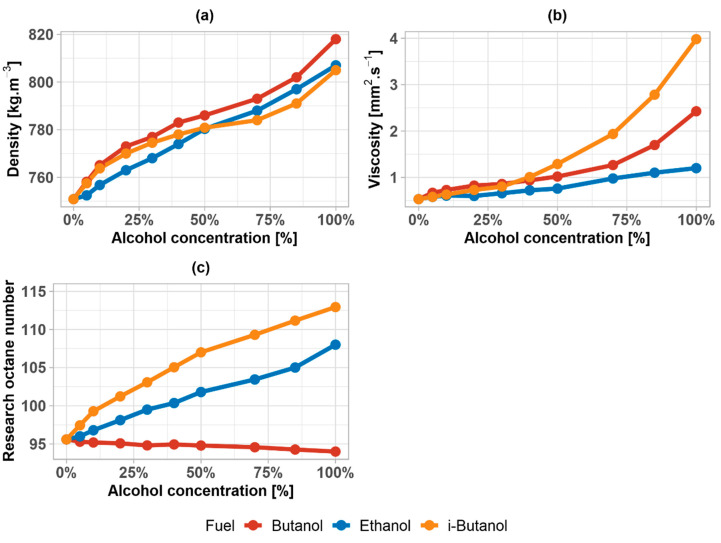
(**a**) Density of ethanol, n–butanol, and i–butanol gasoline mixtures; (**b**) viscosity of ethanol, n–butanol, and i–butanol gasoline mixtures; and, (**c**) research octane number of ethanol, n–butanol, and i–butanol gasoline mixtures.

With the addition of butanol in gasoline, the density and viscosity increase, and the octane number decreases (Figure 1). From this point of view, for example, a fuel consisting of 85% (V/V) of butanol, which is similar to E85 fuel, does not meet the quality requirements according to prEN 15293, but it complies with the BS EN 15293:2018 standard for E85 [74].

The density of fuel mixtures does not differ much in the whole range of interval. The viscosity of alcohols generally increases with increasing number of carbons and the position of -OH group, which is noticeable on the viscosity trend. Ethanol and i–butanol increase the octane number, but n-butanol decreases the octane number due to its carbon chain structure.

### 3.2. Distillation Properties

The following results of the experiments provide the operating parameters of individual alternatives with the accuracy that is given in the methodology. Distillation is a primary process widely used in the oil and petrochemical industries to carry out the fractionation of a feed, providing important qualitative and quantitative information on complex mixtures. From the distillation, a curve is obtained, which is the representation of the boiling temperature of the liquid mixture versus the accumulated volume of distillate at a given pressure. In addition to the distillation curve trend, it is also necessary to focus on a few important values.

- Start of distillation:

Boiling points of the lightest hydrocarbons.

Main cause of fuel losses by evaporation during pumping and storage.

- Ten percent point:

The temperature at which 10% of the fuel volume is distilled.

Expresses the ability of the fuel in order to generate a sufficient proportion of vapors in cold temperatures. A temperature below 80 °C is stated as satisfactory. It is usually around 65–70 °C for current fuels.

- Fifty percent point:

The temperature at which 50% of the fuel volume is distilled.

If this point is above 140 °C, the engine reacts slowly to acceleration. It is usually around 95–115 °C for current fuels.

This point represents the rate of engine heating, which effects the period of time after starting a cold engine and its thrust and power.

- End of distillation curve:

The temperature at which 95% of the fuel volume (or 97% for the fuels with a boiling point higher than 200 °C) is distilled.

These hydrocarbons condense on the cylinder wall during combustion in the engine, where they dissolve the oil layer. This effect, which is referred to as oil dilution, is dangerous due to the low “viscosity reserve” of modern oils.

The value of this point should not exceed 175–180 °C.

Fuel fractions that are above this point have usually the lowest octane number.

- End of distillation temperature:

It is limited to a maximum of 210 °C according to the standard.

Limiting the temperature ensures that all fuel in the engine burns and heavy liquid residues will not dilute the lubricating oil.

Residuals with a boiling point above 200 °C generally do not evaporate, even in a hot engine. They remain in the form of droplets, which only partially burn. The droplets are ejected on the cylinder wall by a vortex in the combustion chamber, dissolves in the oil layer, and thus reduces its viscosity. Heavy end fractions are also involved in the formation of deposits in the combustion chamber, the failure rate of spark plugs, and the formation of resins.

**Figure 2:**
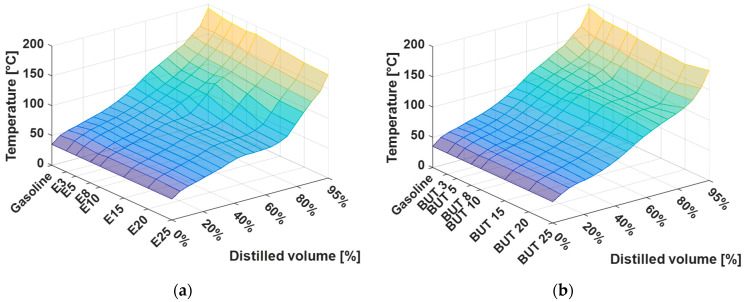

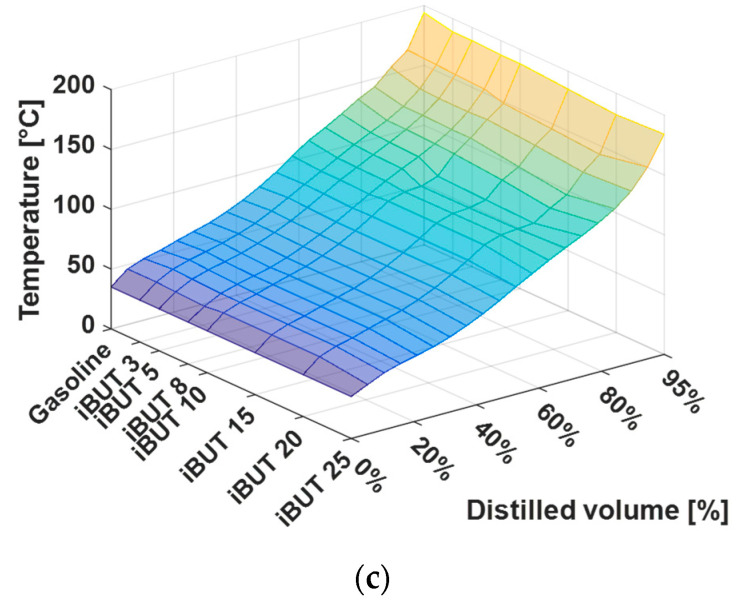
(**a**) Distillation curve of gasoline ethanol mixture; (**b**) distillation curve of gasoline butanol mixture; and, (**c**) distillation curve of gasoline i-butanol mixture.

The distillation curves presented in Figure 2 are a function of temperature of the distilled volume and the temperature difference from gasoline on the distilled volume. The calues were measured per 5% of evaporated volume for three different alcohol-gasoline mixtures:

- E0 (pure gasoline), E3, E5, E8, E10, E15, E20, and E25.
- BUT 0 (pure gasoline), BUT 3, BUT 5, BUT 8, BUT 10, BUT 15, BUT 20, and BUT 25.
- iBUT 0 (pure gasoline), iBUT 3, iBUT 5, iBUT 8, iBUT 10, iBUT 15, BUT 20, and iBUT 25.

From the distillation profile, some specific characteristics of gasoline fuel performance could be evaluated.

The plateau regions in 3D surface graphs show the influence of individual alcohol, which is partially caused by the difference in the boiling points of ethanol, n-butanol, and i-butanol against the gasoline. For the ethanol-gasoline mixture is the temperature drop of around 50% to ~80% of the distilled volume. For the butanol-gasoline mixture is the temperature drop around 70% to ~90% of the distilled volume. For the i–butanol-gasoline mixture is the temperature drop around 60% to ~90% of the distilled volume. This drop is also caused by the non-ideality of the liquid, because of the formation of a near-azeotropic mixture. By evaporating the alcohol, the near-azeotropic mixture is removed and the distillation temperature increases toward that of the remaining gasoline hydrocarbons.

**Figure 3:**
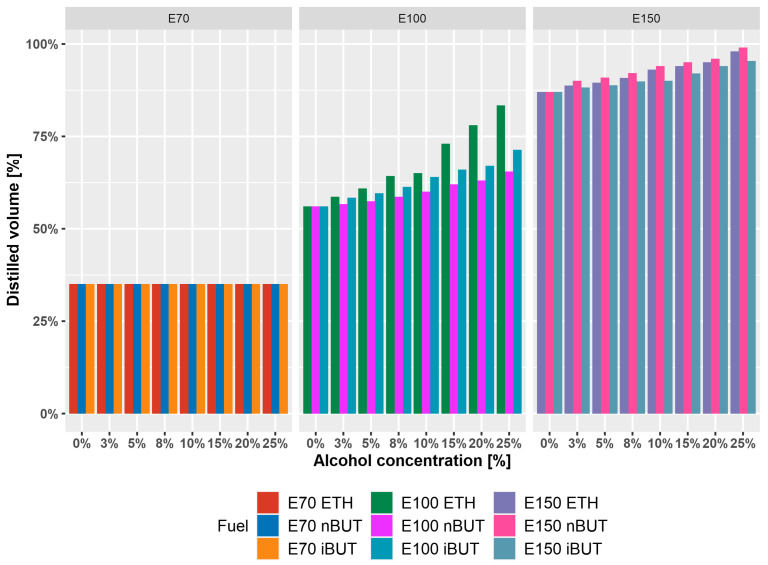
Influence of alcohol content on E70, E100, and E150 of alcohol-gasoline mixture.

Figure 3 shows the differences in the distilled volume at 70 °C (E70), 100 °C (E100), and 150 °C (E150), depending on the volume of added alcohol. All of the alcohols have a boiling point above 70 °C, and only the light components of gasoline are evaporating. As boiling point is increasing (ethanol–78.5 °C, i–butanol–107.89 °C, n–butanol–117.7 °C), the effect and trend on T100, and T150 are affected, respectively.

**Figure 4:**
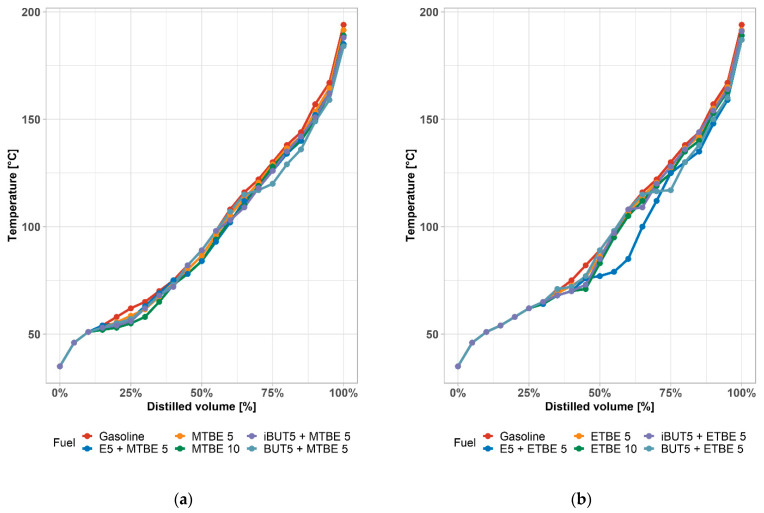
(**a**) Distillation curves of gasoline containing ethanol, n–butanol, and i–butanol with Methyl tert-butyl ether (MTBE); (**b**) distillation curves of gasoline containing ethanol, n–butanol, and i–butanol with ethyl tert-butyl ether (ETBE).

Figure 4a,b shows the effect of mixing MTBE and ETBE cosolvents to the analyzed mixtures. These ethers also affect the shape of the distillation curve and they are well recognizable. Ethers affect the middle part of the distillation curve most—around 10–85%. In the case of the combination of 5% (V/V) ethanol and 5% (V/V) ETBE, the effects of these oxygen compounds on the change in the distillation curve profile add up, which does not apply to mixtures with butanol. Changes can be seen in the distillation curve against pure gasoline by the decrease of distillation in a wide range of distilled volume (30–85%) or in a wide range of distillation temperatures (50–150 °C). Higher concentrations of alcohols and ethers (more than 10% (V/V) of each) also cause significant changes in the region of the second half of the distillation curve.

### 3.3. Vapour Pressure

**Figure 5:**
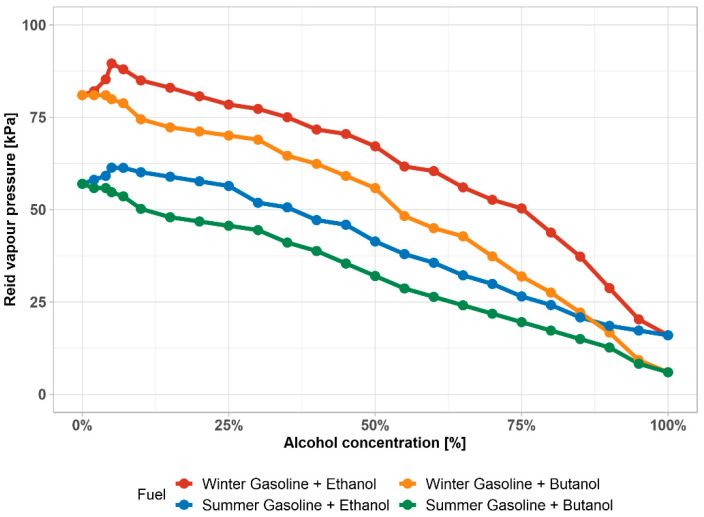
Reid vapor pressure of winter and summer gasoline mixtures, depending on the volume of alcohol added.

Alcohol–gasoline mixture as compared to pure gasoline generally has lower Reid vapor pressure (RVP—vapor pressure at 37.8 °C (100 °F)). The winter gasoline used in these analyses had an RVP = 87 kPa and summer gasoline had an RVP = 57 kPa. Figure 5 shows the results of the Reid vapor pressure in kPa of the summer and winter gasoline with different alcohol content.

Figure 5 plots the vapor pressure of the winter and summer gasoline differences for the alcohol–gasoline mixtures. Ethanol has a higher influence on RVP against butanol, but. overall, the trend is decreasing. However, addition of 5% (V/V) of ethanol to gasoline actually leads to an increase of RVP for both winter and summer gasoline. This is caused by the formation of ethanol azeotrope with aromatic compounds. Butanol, on the other hand, does not form azeotropes much. The RVP of ethanol–gasoline mixture equalizes with pure gasoline around 20–25% (V/V).

Ethanol and butanol both have the same influence in winter mixtures and summer mixtures up to ~75% (V/V) and ~55% respectively. Above these concentrations, the influence is increasing, and RVP is more rapidly decreasing.

**Figure 6:**
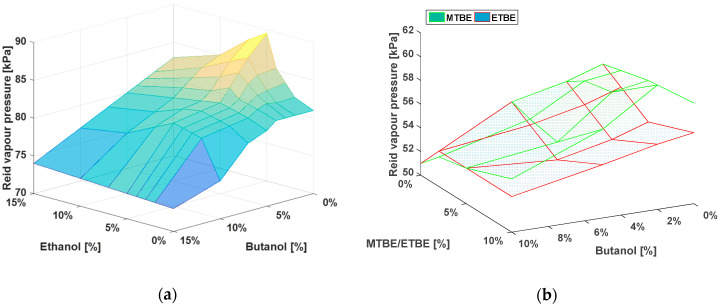
(**a**) Reid vapor pressure of winter gasoline mixtures depending on the volume and type of added alcohol; (**b**) Reid vapor pressure of a summer gasoline with n-butanol, MTBE, and ETBE.

Figure 6a depicts the influence of volumetric composition (V ethanol + V n‑butanol in gasoline) among alcohol mixtures and gasoline. There is a clear global maximum in 5% (V/V) of ethanol and 95% (V/V) of gasoline, where the aforementioned positive azeotrope is formed. In order to reduce the vapor pressure of the azeotrope in 5% (V/V) ethanol mixture, a small amount of n-butanol can be added so the desirable properties are met. It can obtain approximately the same RVP as pure gasoline by adding butanol to only 5/5% (V/V) mixture. The surface plot also reveals that 5/10% (V/V), 10/5% (V/V), and 2/5% (V/V) mixtures have the same RVP as pure gasoline.

Figure 6b depicts the comparison between MTBE and ETBE. ETBE has a more favorable effect on mixtures with butanol, because it has a lower effect on the vapor pressure of mixtures. Even though their mixed vapor pressure is effectively the same as the vapor pressure of pure substances, ETBE reduces the vapor pressure in the whole range of gasoline RVP i.e., 45–90 kPa, the influence of MTBE depends on the initial vapor pressure of the hydrocarbon base. For values that are below 55 kPa, MTBE increases the vapor pressure and, above it, MTBE decreases the vapor pressure. This was also the reason summer gasoline was chosen for the performed analysis.

Figure 6 generally shows that the addition of 5% (V/V) of ethanol in a mixture increases the RVP the most and any change of this composition results in a decrease of RVP. The rate of RVP decreasing in the analyzed range is actually when both ethanol and n-butanol are added in the same volumetric ratio ~1 kPa per 1% (V/V).

### 3.4. Water Stability

Hydrocarbons in gasoline are very slightly miscible with water in contrast to alcohols. The mixtures of gasoline with alcohols have a limited ability to retain water in solution or in a stable emulsion. If the amount of water exceeds the water solubility limit, the fuel will be separated into two immiscible phases—aqueous and hydrocarbon. The solubility of water in pure hydrocarbon gasoline varies depending on the content of aromatics in the range of 60–100 mg/kg. Ethanol is completely miscible with water, 1‑butanol is only 73 g/L—see Table 1 for the water solubility for all butanol isomers.

**Figure 7:**
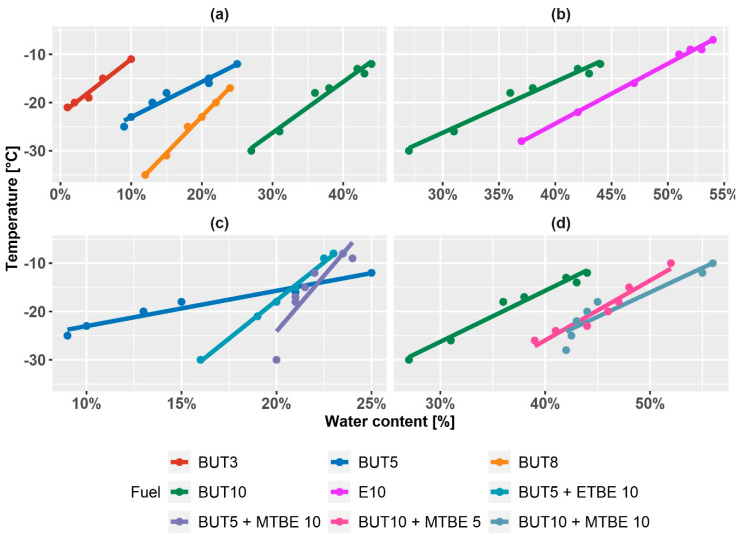
The solubility of water in alcohol-gasoline blends expressed as the temperature of crystallization. (**a**) Comparison of butanol-gasoline mixture with increasing volume of butanol in mixture; (**b**) comparison of 10% (V/V) n–butanol and 10% (V/V) ethanol; (**c**) comparison of 5% (V/V) n–butanol with addition of 10% (V/V) MTBE and 10% (V/V) ETBE; (**d**) comparison of 10% (V/V) n‑butanol with the addition of 5% (V/V) MTBE and 10% (V/V) MTBE.

The solubility of water in fuel decreases linearly with decreasing temperature. The determination of water solubility according to ASTM D6422 is based on this phenomenon. Water solubility in alcohol–gasoline mixture from three measurements differed by less than 1 °C. The expanded uncertainty of the result determination is ±1 °C. See the measured solubility in Figure 7.

The results depicted in Figure 7 show a linear relation between temperature and water content. For example, fuel containing 10% (V/V) of butanol can absorb ~2.3 times more water than fuel containing 5% (V/V) of butanol. All of the data points were correlated to a linear function. Table 3 presents the parameters of each correlation.

**Figure 8:**
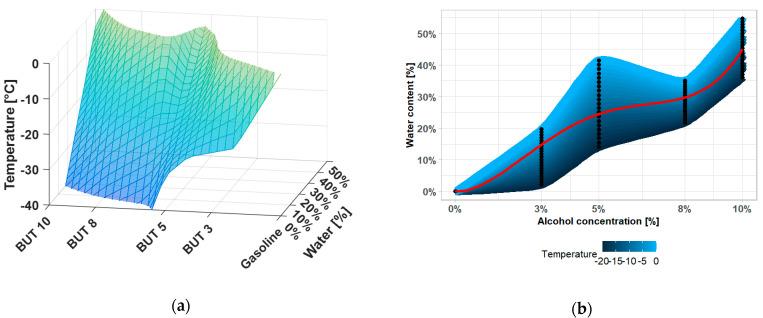
(**a**) Surface plot of nonlinear relation among the temperature, water content, and n-butanol-gasoline mixture composition; (**b**) projection of surface plot to two-dimensional (2D) gradient plot of nonlinear relation among the variables.

Increasing the volume of alcohol-gasoline mixture influences the increase of the water solubility in the fuel in a nonlinear way. In Figure 8, see where the dependence of water solubility on temperature and n-butanol content is depicted. This property is shared among all of the alcohol-gasoline blends. The red line approximates the mean trend in one regression curve. The measurements show that the solubility of water in a mixture with 10% (V/V) of butanol at 0 °C is approximately 5400 mg/kg. In the case of a mixture with 10% (V/V) ethanol, it is approximately 5900 mg.kg^−1^.

Ethers in the amounts of 5% and 10% (V/V) alongside 5% and 10% (V/V) of butanol were also measured. The effect of some ethers on the phase stability of the mixtures is also significant. In the case of ETBE, the phase separation was found and, i.e., the precipitation of the aqueous phase in the form of crystals occurred. The presence of MTBE, on the other hand, does not significantly affect the solubility of water in the mixture. Gasoline containing both butanol and ether at the same time behaves similarly to the butanol–gasoline mixture in terms of phase stability. Thus, there was no visible clouding followed by phase separation. If any, the present water precipitated directly in the form of ice crystals.

### 3.5. Gas Chromatography Analysis

The gas chromatography procedure evaluated both n-butanol and i–butanol while separating the peaks of fractions C_5_, C_6_, and mobile phase (isooctane). The internal standard was correctly selected because the visible peak in the chromatogram was a sufficient distance from the analytes. The selected nonane met this requirement well. The conditions for chromatographic separation were also fulfilled, since both n-butanol and i–butanol were separated from the other parts of the sample. The minimal differences between the retention times of the analytes show a very good reproducibility of the retention time.

**Figure 9:**
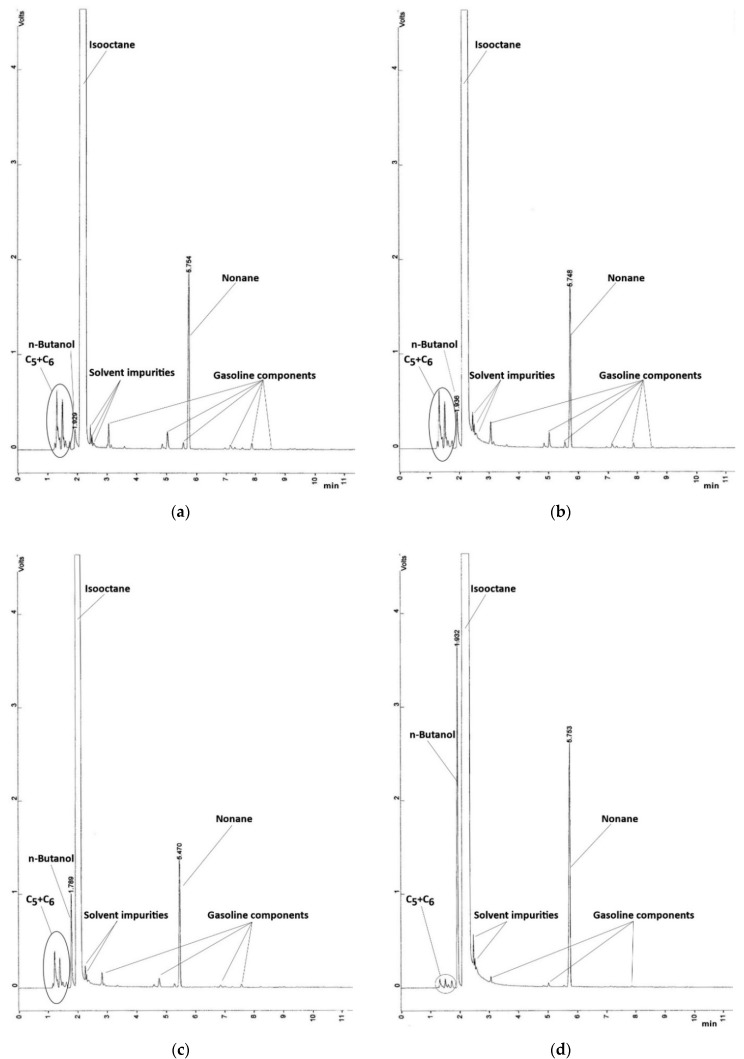
(**a**) Chromatogram of BUT 5 mixture; (**b**) chromatogram of BUT 10 mixture; (**c**) chromatogram of BUT 30 mixture; (**d**) chromatogram of BUT 85 mixture.

Figure 9a–d depict chromatograms of n-butanol-gasoline mixtures BUT 5, BUT 10, BUT 30, and BUT 85, respectively. The interval 0−11 min. in retention time was selected according to the evaluated substances. Other components in the mixture were not relevant for the analysis of the n-butanol and i–butanol. The peak of the isooctane in the solvent was cut off to capture a greater detail of analyzed alcohols.

The same measurement was also conducted for mixtures iBUT 5, iBUT 10, iBUT 30, BUT 85, BUT 5 + iBUT 5, and BUT 10 + iBUT 10 with equally conclusive results in the chromatograms. Measured concentration of each alcohol in the mixtures is presented in Table 4.

## 4. Discussion

Biobutanol holds particular promise as a liquid transportation fuel, with advantages over bioethanol as part of a wider portfolio of sustainable energy solutions. The experiments showed that the biobutanol is a suitable alternative to widespread bioethanol and, in many properties, exceeds it. The use of both n-butanol and i–butanol in transport is the subject of research by many scientific groups around the world [38,41,43,44,45,46,47,48,52,59,60,61,62,75,76,77,78]. This paper contributes to this research by providing a comprehensive view into several aspects of fuel mixtures all together.

It was measured that butanol has a higher kinematic viscosity value than gasoline and ethanol, and the viscosity of the mixtures thus increase with the addition of butanol. The use of butanol in high-percentage mixtures could thus cause greater stress on the fuel system, which is also related to the increased density of the mixtures. Da Silva et al., 2005 [79] analyzed the viscosity of ethanol, MTBE, ETBE mixtures (among others), with two different gasolines with different compositions. The values measured by them were higher (against this paper) by 15–20% for both ethanol–gasoline and n-butanol–gasoline mixture.

The octane number of gasoline–alcohol mixtures is greatly influenced by the octane numbers of the individual alcohols. Butanol was expected to reduce the octane number in gasoline, which was also verified. However, even with high-percentage mixtures of butanol in gasoline, the decrease is not so significant that it could affect the combustion and anti-knock resistance of the fuel, and it essentially respects the requirements that are set by standards. One of the possibilities for influencing the anti-knock properties could be the in situ production of alcohols in gasoline, which could potentially replace ether oxygenates [80] or the use in combination with octane booster additives, for example, MTBE (RON = 118), ETBE (RON = 118), 1,3,5-trimethylbenzene (RON = 137), propylbenzene (RON = 129), m-xylene (RON = 124), etc. [81,82]. Corrubia et al., 2020 [83], Da Silva et al., 2005 [79], or Lapuerta et al., 2017 [84] measured the research octane number for ethanol, n-butanol, and i-butanol. The results of our research complement the results of these authors by analyzing mixtures while using gasoline from Central Europe. The results correspond qualitatively, and the differences are only caused by differences in the gasoline used.

Distillation curves are characterizing the boiling points of individual hydrocarbons and they predict the behavior of the fuel from the injection process to combustion. The addition of an alcohol to gasoline significantly affects the boiling point of mixture. Ethanol mainly affects the first half of the distillation curve and, especially, the temperature T50 (50% of volume is distilled), due to the near-azeotropic behavior. Butanol affects the second half of the distillation curve, which is also reflected in the values of E100 and E150. These are important both in terms of engine operation and in terms of quality parameters. The amount of distilled volume up to 100 °C that was characterized by the values of E70 and E100 (distilled volume at 70 °C and 100 °C) influences the cold start of the engine. Experiments were also focused on the evaluation of the distillation curve of mixtures in the presence of commonly used cosolvents MTBE and ETBE. Ethers also significantly affected the trend of distillation curves. Cosolvents affect the middle part of the distillation curve the most and, in the case of a joint mixtures of ethers and ethanol, the effects on the curve add up. However, this problem does not apply when ethanol is replaced by butanol in the form of n-butanol or i–butanol. Da Silva et al., 2005 [79] also analyzed the distillation parameters of mixtures and results corresponded qualitatively to the distillation curve found here. The same effect of ethanol azeotrope occurred in 5% (V/V) concentration. Shirazi et al., 2019 [85] analyzed methanol, ethanol, i–butanol, 3–methyl–3–pentanol, and their mixtures. When compared to our results, the distillation temperatures for all blends differ by ±10%, which is qualitatively the same result, being affected by a different base gasoline. They concluded the same that the mixtures have satisfactory properties for use in existing spark ignition engines. Andersen et al., 2010 [86] also presented the distillation curves for single-alcohol mixtures in gasoline, containing 5–85% (V/V) of methanol, ethanol, n–propanol, i–propanol, n–butanol, s–butanol, i–butanol, and t–butanol. Most of the distillation curves have the qualitatively same values and trend for the most important points on the distillation curves (T0, T10, T50, T90). In some cases, T50 were lower than the results in this paper, because of the different base gasoline. Similar results were published also by Amine et al., 2020 [87] (T50 ≈ 75 °C), Castillo-Hernández et al., 2012 [88] (T10 = 46.6 °C and 48.1°C, T50 = 65.3 °C and 68.7 °C, T90 = 136.5 °C and 142.2 °C), and many more [89,90,91]. As before, differences in results are there due to different base gasoline being used in individual analyses.

Reid vapor pressure is used to characterize the volatility of gasolines and it is the industry standard measure for vapor pressure. Low RVP fuels are associated with cold start problems and higher soot production, while high RVP fuels are associated with vapor lock [92]. Vapor pressure can be regulated by additives or it is possible to use winter gasoline, which evaporates more than the summer gasoline. Gaspar et al., 2019 [93] analyzed Reid vapor pressure for methanol, ethanol, n–propanol, and i–propanol for various concentrations in gasoline. The results are similar to summer gasoline mixtures analyzed in this paper. The difference can be found in the longer manifestation of azeotrope, where RVP decreases slowly. The influence of azeotrope in ethanol-gasoline mixture is a well-known phenomenon [86,94,95,96,97]. The RVP ranges between 52–60 kPa for ethanol-gasoline mixture and 52–20 kPa for n-butanol-gasoline mixture. These results are qualitatively same with same trend. In this paper, RVP ranges between 51–43 kPa for ethanol-gasoline mixture and 51–16 kPa for n-butanol-gasoline mixture. Da Silva et al., 2005 [79] also analyzed RVP of the mixtures and they can be assessed as qualitatively the same for both of the gasolines in the evaluated range—in measured range 0–25% is RVP different at most by 2 kPa (~3.5% difference).

For the usability of the fuel, an analysis of its low-temperature parameters and the ability of water-solubility is also important parameter. The safe water content, which should be dissolved in gasoline containing 5% (V/V) of ethanol under summer and winter conditions, should not exceed 0.02% (m/m). (200 ppm). The negative effect of gasoline-ethanol fuels is that the layers separate, and the alcohol passes into the aqueous phase during separation. Although the solubility of water in gasoline–butanol and gasoline–ethanol mixtures is very similar, ethanol is completely miscible with water, but butanol is only slightly miscible with water. At low temperatures, the mixture gradually becomes cloudy up to the point where water is precipitated to form crystals. Butanol does not pass into the aqueous layer as compared to ethanol. Water solubility was reported also by Mužíková et al. 2012 [98], where the results differ up to ±0.1% (m/m). This topic was also discussed in Gramajo de Doz et al. 2004 [99] for higher temperatures.

An analytical method for the determination of butanol in gasoline using GC-FID was also developed and validated. GC-FID analysis provided characteristic records for gasoline fractions with different concentration of ethanol, n–butanol, and i–butanol. The analysis correctly detected the presence of components. The use of the GC-FID method for measuring the fuel composition was also used by Yoram Gerchman et al., 2012 [100] or Lin et al., 2014 [101].

Gasoline engines can burn mixtures of gasoline with butanol that formed in any ratio of the two components, but it can also be used separately (100%) as a propellant in internal combustion engines. If the mixture should comply with the conditions of EN 228:2008, allowing for the oxygen content in gasoline up to a maximum of 2.7% (m/m), it would be possible for gasoline to mix up to 11.7% (V/V) of butanol and, in the future, up to 16% (V/V), without engine modification; in the case of ethanol mixing, it is only a maximum of 5% (V/V). Butanol is not as hygroscopic as ethanol, so it is less corrosive to metal tanks and pipes, nor does it create separate water phase (it behaves similarly to MTBE or ETBE ethers). Therefore, it can be transported by conventional and already existing pipelines, tanks, and only distributed by partially modified filling stations, which practically excludes the transport of ethanol in existing facilities. From the point of view of handling, butanol is also safer than ethanol fuel due to lower vapor pressure [39,40,42,43,45,98].

## 5. Conclusions

This paper investigates various physicochemical properties of n-butanol and i–butanol in comparison with widespread bioethanol. Properties, such as viscosity, density, octane number, vapor pressure, distillation characteristics, or water solubility, were thoroughly discussed. The main discoveries are summarized below:

- Butanol has a higher kinematic viscosity value than gasoline and ethanol. The viscosity of the mixture increases with the addition of butanol.
- The octane number of mixtures is mainly influenced by the octane numbers of the individual alcohols. Butanol reduces the octane number in gasoline, but the decrease is not so significant.
- Ethanol with gasoline creates an azeotrope, which increases the vapor pressure of formed mixture. Such a phenomenon does not occur with butanol.
- Ethers can have a positive effect on the increase of vapor pressure. It was verified that ETBE has more favorable effects than MTBE.
- Ethanol mainly affects the first half of the distillation curve and especially the temperature T50. Butanol mainly affects the second half of the distillation curve, which is also reflected in the values of E100 and E150.
- The solubility of water in butanol and ethanol mixtures is similar. However, butanol is more stable at low temperatures. Water is only slightly soluble in MTBE and ETBE.

## Figures and Tables

**Table 1:** Comparison of the properties of butanol isomers with other conventional fuels [59,60,61,62,63].

Properties	Diesel	Gasoline	Ethanol	n–Butanol	s–Butanol	i–Butanol	t–Butanol
Molecular weight	198.4	111.19	46.07	74.11	74.11	74.11	74.11
Cetane number	40–55	0–10	5–8	12.0	8.5	8.5	5.6
Research octane number	20–30	91–99	108	94	101	113	105
Motor Octane Number	–	81–89	89–103	78	91	94	89
Density [g/mL] at 20 °C	0.82–0.86	0.72–0.78	0.789	0.808	0.808	0.805	0.800
Flash point [°C]	65–88	−45 to −38	14	35	34	24	28
Water solubility at 25 °C [g/L]	0.01–0.06	0.046–0.077	∞	73	185.1	89.4	∞
Boiling point [°C]	180–370	25–215	78.5	117.7	99.51	107.89	82.4
Flammability [% (V/V)]	1.5–7.6	0.6–8	3.3–19	1.4–11.2	1.7–9.8	1.7–10.9	2.3–8
Reid vapor pressure [kPa]	0.2–0.7	75	16.5	6	5.3	3.3	12.2
Viscosity [mm^2^/s] at 25 °C	1.9–4.1	0.4–0.8	1.07	2.63	3.1	4	4.31
Energy density [MJ/L]	35.86	32	25	29.2	29.06	29.00	28.48

In case of infinity “∞” solubility, it means that alcohol is fully miscible in water.

**Table 2:** Measured parameters of mixtures of n-butanol with gasoline.

Parameter	Unit	BUT 5	BUT 30	BUT 50	BUT 85	BUT 100
Density at 15 °C	kg∙m^−3^	736.16	781.5	786.01	802.95	813.57
Start of distillation	°C	34	33	35	40	117
Evaporated vol. at 70 °C	% (V/V)	36	32	22	*	*
Evaporated vol. at 100 °C	% (V/V)	57	55	45	12	*
Evaporated vol. at 150 °C	% (V/V)	89	*	*	*	*
End of distillation	°C	188	117	117	117	117
Distillation residue	% (V/V)	1.2	1.8	2.3	3.2	4.5
Reid vapor pressure	kPa	48.0	45.5	30.5	26.0	8.1
Volatility index	–	725.0	679	459	**	**
Viscosity at 40 °C	mm^2^∙s^−1^	0.67	0.86	1.02	1.69	2.43
Flash point	°C	**	**	**	**	35

* Value does not exist. ** Parameter cannot be evaluated according to valid standards for liquid fuels and petroleum products.

**Table 3:** Parameters *A* and *B* of the linear equation *T = Aw + B* depicted in Figure 7, correlation coefficients, R^2^, and *p*-value for each fit of water solubility of fuel mixtures.

Fuel	*A*	*B*	R^2^	*p*-Value
BUT 3	114.5	−22.5	0.966	1.90 × 10^−3^
BUT 5	73.1	−30.3	0.951	1.17 × 10^−4^
BUT 8	151.3	−53.1	0.993	1.02 × 10^−5^
BUT 10	107.8	−58.9	0.984	4.26 × 10^−14^
E10	120.8	−72.6	0.984	1.06 × 10^−8^
BUT 5 + MTBE 10	315.5	−80.8	0.755	6.96 × 10^−3^
BUT 5 + ETBE 10	460.7	−116.3	0.995	3.98 × 10^−7^
BUT 10 + MTBE 5	123.8	−75.5	0.930	2.83 × 10^−4^
BUT 10 + MTBE 10	101.2	−66.6	0.845	2.13 × 10^−3^

**Table 4:** Measured concentrations of n-butanol and i–butanol in gasoline.

Mixture	Measured Concentration of Alcohol [% (V/V)]	
BUT 5	6.08	
BUT 10	9.50	
BUT 30	33.88	
BUT 85	82.92	
iBUT 5	4.46	
iBUT 10	9.76	
iBUT 30	34.11	
iBUT 85	83.12	
BUT 5 + iBUT 5	6.73	5.11
BUT 10 + iBUT 10	8.01	7.06

## Data Availability

Data is contained within the article.
